# Palliative care referral criteria and outcomes in cancer and heart failure: a systematic review of literature

**DOI:** 10.1186/s40959-021-00117-8

**Published:** 2021-09-23

**Authors:** Anecita P. Fadol, Ashley Patel, Valerie Shelton, Kate J. Krause, Eduardo Bruera, Nicolas L. Palaskas

**Affiliations:** 1 Department of Nursing, The University of Texas MD Anderson Cancer Center, Houston, TX USA; 2 Department of Cardiology, The University of Texas MD Anderson Cancer Center, Houston, TX USA; 3 Department of Internal Medicine, Baylor College of Medicine, Houston, TX USA; 4 Research Medical Library, The University of Texas MD Anderson Cancer Center, Houston, TX USA; 5 Department of Palliative, Rehabilitation and Integrative Medicine, The University of Texas MD Anderson Cancer Center, Houston, TX USA

**Keywords:** Palliative care, Supportive care, cancer, Heart failure, Referral criteria, Outcomes

## Abstract

**Background:**

Cardiotoxicity resulting in heart failure (HF) is among the most dreaded complications of cancer therapy and can significantly impact morbidity and mortality. Leading professional societies in cardiology and oncology recommend improved access to hospice and palliative care (PC) for patients with cancer and advanced HF. However, there is a paucity of published literature on the use of PC in cardio-oncology, particularly in patients with HF and a concurrent diagnosis of cancer.

**Aims:**

To identify existing criteria for referral to and early integration of PC in the management of cases of patients with cancer and patients with HF, and to identify assessments of outcomes of PC intervention that overlap between patients with cancer and patients with HF.

**Design:**

Systematic literature review on PC in patients with HF and in patients with cancer.

**Data sources:**

Databases including Ovid Medline, Ovid Embase, Cochrane Library, and Web of Science from January 2009 to September 2020.

**Results:**

Sixteen studies of PC in cancer and 14 studies of PC in HF were identified after screening of the 8647 retrieved citations. Cancer and HF share similarities in their patient-reported symptoms, quality of life, symptom burden, social support needs, readmission rates, and mortality.

**Conclusion:**

The literature supports the integration of PC into oncology and cardiology practices, which has shown significant benefit to patients, caregivers, and the healthcare system alike. Incorporating PC in cardio-oncology, particularly in the management of HF in patients with cancer, as early as at diagnosis, will enable patients, family members, and healthcare professionals to make informed decisions about various treatments and end-of-life care and provide an opportunity for patients to participate in the decisions about how they will spend their final days.

## Introduction

Cancer and heart disease are the leading causes of death in the United States [1]. The intersection of both is addressed by a new multidisciplinary specialty known as cardio-oncology, which focuses on cardiovascular care in patients with cancer. Among the most dreaded complications of cancer therapy is heart failure (HF), which can occur acutely during the therapy or arise several years after completion of the therapy. The burden of disease and its associated impact on the patient and caregiver in cancer and HF are exceedingly high and compounded when both diseases coexist. An interdisciplinary palliative care (PC) intervention can improve the patient’s quality of life, while minimizing caregiver distress and aggressive measures at the end of life. The World Health Organization (WHO) defines PC as “an approach that improves the quality of life of patients and their families, facing the problem associated with life threatening illness, through the prevention and relief of suffering by means of early identification and impeccable assessment and treatment of pain and other problems, physical, psychosocial and spiritual.” The WHO recommends that PC should be available to everyone suffering from life-threatening diseases and should be started early in the illness trajectory [2].

In oncology patients, one of the key barriers to early PC referral is the misunderstanding that PC is only provided at the end of life once patients have exhausted all cancer treatment options. Oftentimes, PC is misinterpreted for hospice or end of life care. Therefore, it is crucial to differentiate between PC and hospice care. Hospice is comfort care without curative intent and is used when the patient no longer has curative options or attempts to cure the person’s illness are stopped and the individual is approaching the end of life [3]. Whereas in PC, patients may receive medical care for their symptoms, along with treatment intended to cure their serious illness. PC is meant to enhance a person’s current care by focusing on quality of life for them and their family.

The traditional model of PC is a system of care delivery most appropriate for patients with a predictable trajectory of illness and death, such as that of terminal cancer. Similar to cancer, the advancement of HF into later stages also follows known patterns as symptoms become more intense and refractory to standard treatments, leading to recurrent acute-care utilization and contributing to poor quality of life [4]. However, the symptom burden and HF classification, such as the New York Heart Association (NYHA) classification [5], are dependent on a patient’s fluid overload status and can wax and wane unpredictably. Regardless, the symptom burden in HF, including dyspnea, pain, anxiety, fatigue, and depression, can equal or exceed that in cancer populations [6]. Yet, evidence shows that HF patients have suboptimal access to and provision of PC and hospice [7–11].

In 2015, the National Academy of Medicine (formerly called the Institute of Medicine) issued recommendations to improve advance care planning and increase access to PC for all seriously ill patients [12, 13]. Subsequently, leading professional societies including the American College of Cardiology, American Heart Association, Heart Failure Society of America, and the International Society for Heart and Lung Transplantation published clinical guidelines recommending improved access to hospice and PC for patients with advanced HF [14–20]. Likewise, the American Society of Clinical Oncology, the National Comprehensive Cancer Network (NCCN), and the National Academy of Medicine have endorsed timely PC referral for cancer patients [21–23]. However, despite guidelines recommending the inclusion of PC, there are limited data offering guidance on PC in patients with a dual diagnosis of cancer and HF. Therefore, we conducted this systematic literature review with the following aims:

1. To identify existing criteria for referral to and early integration of PC in the management strategies for patients with cancer and patients with HF.
2. To identify assessments of outcomes of PC intervention that overlap between patients with cancer and patients with HF.

This review will provide baseline information to define best practices for referral to and successful delivery of PC to patients living with cancer and HF.

## Methods

### Study design and search strategy

We performed a systematic search of the literature for studies assessing criteria for and outcomes of PC referral in both HF and cancer. We searched Ovid Medline, Ovid Embase, Cochrane Library, and Web of Science from January 2009 to September 2020. Search structures, subject headings, and keywords were tailored to each database by a medical research librarian (KJK) specializing in systematic reviews. Case reports, animal studies, and articles in languages other than English were excluded, without any other restrictions by study type. Search strings included MeSH and Emtree subject headings, which included: “heart failure”, “neoplasms”, “hospice care”, and “palliative care”. Keyword searching was used to retrieve articles with related terms and phrases in the titles and abstracts.

### Study selection

Our initial search retrieved 8,647 citations, and after removal of duplicates, 5,482 citations remained for review, comprising 4,180 articles for cancer and 1,302 articles for HF. Citations were independently screened by two investigators (APF, AP) by using the titles and abstracts of the articles to identify potentially relevant studies. Disagreements were resolved by consensus and by seeking the opinion of a third reviewer (NLP). Studies that passed the title/abstract review were retrieved for full-text review. The two screening investigators (APF, NLP) then independently screened the remaining full-text articles. Disagreements were resolved by consensus and by seeking the opinion of a third reviewer (EB). After final review, 16 studies on cancer and 14 studies on HF were included. A PRISMA flow diagram (

**Fig. 1:**
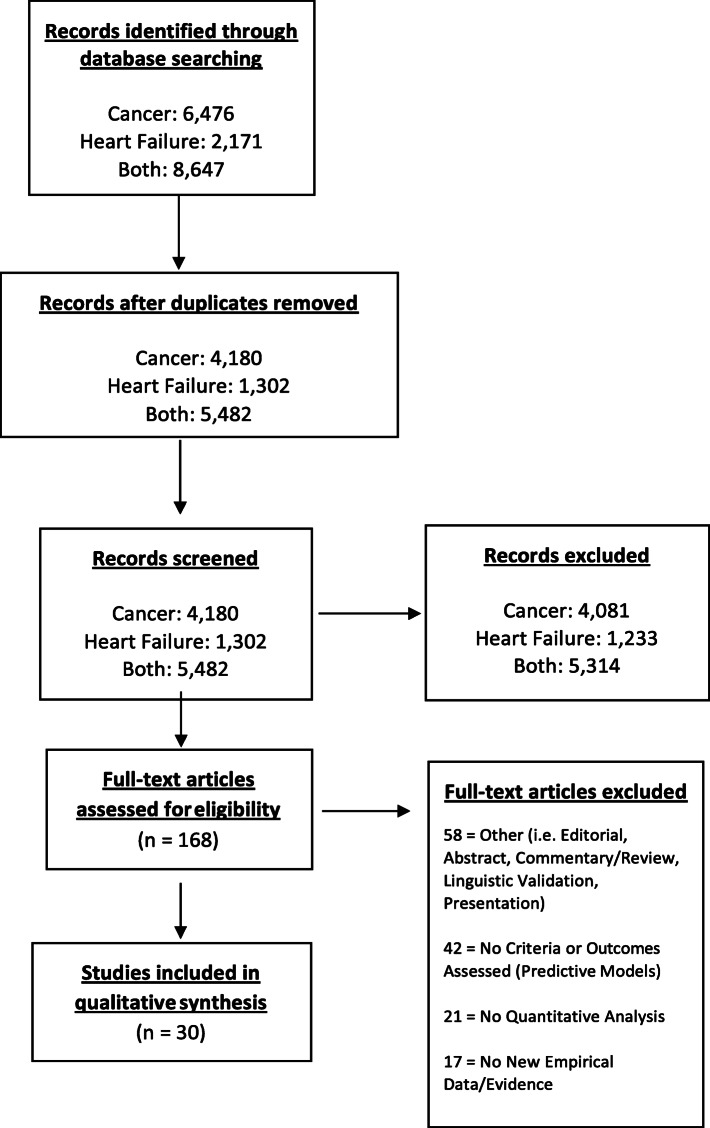
PRISMA diagram

Figure 1) shows the entire review process from the original search to the final selection of studies.

### Statistical methods

The main outcome measures for this systematic literature review were criteria for and outcomes of referral to PC for patients with diagnoses of cancer and HF. Because of the heterogeneity of study designs, participants, interventions, and reported outcomes, meta-analytical statistical comparison was not possible. Therefore, we focused on describing the studies, their results, and their limitations via a qualitative synthesis.

## Results

The studies selected for inclusion in the review were analyzed for risk of bias to understand and appraise their strengths and weaknesses, and results are outlined in

**Table 1:** Risk of Bias - Cancer

Bias Category	Individual Study															
	Sanders 2010	Grudzen 2010	Temel 2010	Glare 2011.	Glare, 2013	Hui 2014	Bakitas 2015	Rocque 2015	Hui 2016	Adelson 2017	Molin, 2019	Brinkman- Stoppe lenburg 2019	Hui 2020	Caraceni 2020	Gemmel 2020	Hansen 2020
Study Design	M	M	L	M	M	L	L	M	L	M	M	M	H	M	M	M
Selection of non-exposed cohort	M	H	L	M	L	L	L	M	L	H	L	M	M	M	M	M
Bias due to confounding	H	H	L	M	M	L	L	M	L	H	M	M	M	M	M	M
Bias in classification of intervention	M	M	L	L	L	L	L	M	L	M	L	L	M	M	M	M
Bias due to deviations from intended intervention	L	M	L	M	M	M	M	H	NA	M	M	M	M	L	L	NS
Bias due to missing data	M	M	M	M	NS	M	M	M	L	M	NS	NS	NS	M	NS	NS
Bias in measurement of outcomes	M	H	M	M	M	L	L	M	NA	M	L	M	L	M	M	M
Bias in selection of the reported result	M	M	L	L	L	L	L	M	L	M	M	L	L	M	M	M

*L*Low risk of bias; *M* medium risk of bias; *NS* not specified or applicable

Studies shown by author

Table 1 (cancer) and

**Table 2:** Risk of Bias—Heart Failure

Bias Category						Individual Study								
	Harding 2009	James 2010	Ezekowitz 2011	Unroe 2011	Greener 2014	Kheirbek 2015	Rogers 2017	Campbell 2018	Kane 2018	Ng Fat Hing 2018	Liu 2020	Roch 2020	Truby 2020	Avula 2000
Study design	M	H	M	H	H	H	L	M	M	H	H	M	L	H
Selection of non-exposed cohort	H	NS	NS	NS	M	M	L	M	M	M	H	H	L	NS
Bias due to confounding	H	M	M	H	M	M	L	M	M	M	H	H	L	H
Bias in classification of intervention	M	H	M	M	M	M	L	M	M	M	M	M	L	M
Bias due to deviations from intended intervention	L	M	L	M	M	NS	L	M	L	L	L	L	L	L
Bias due to missing data	L	L	L	M	L	L	L	L	L	L	L	L	L	L
Bias in measurement of outcomes	M	M	M	M	M	M	L	M	M	M	L	M	M	M
Bias in selection of the reported result	M	M	M	M	M	M	L	M	M	M	M	M	L	M

*L* low risk of bias; *M* medium risk of bias; *H* high risk of bias; *NS* not specified or applicable

Studies shown by first author

Table 2 (heart failure).

### Cancer

Of the 16 studies included in the systematic review regarding cancer and PC, nine studies looked at referral criteria, and six studies evaluated referral outcomes. Five studies were prospective [24–28], four were retrospective [29–32], five were cross-sectional surveys [33–37], and two randomized controlled trials [28, 38] (

**Table 3:** Palliative/Supportive Care in Cancer Patients

Study	Population	Aims	Design	Key Findings
Sanders, et al. 2010.	109 patients with lung cancer	To characterize the prevalence and intensity of supportive care needs and interest in specific supportive care services among individuals with lung cancer	Cross-sectional survey	Participants reported the greatest need in the physical and daily living domain, followed by psychological needs, health system and informational needs, and patient care support needs. The most common unmet need was a lack of energy and tiredness (75%). Higher levels of supportive care needs were associated with worse physical functioning, greater symptom bother, lower satisfaction with health care, and higher levels of intrusive thoughts about cancer.
Grudzen et al. 2010.	50 seriously ill adults with co- existing cancer in the emergency department	To identify the palliative care needs of seriously ill, older adults in the emergency department (ED).	Cross-sectional survey	Over half of the patients exceeded intratest severity-of- needs cutoffs in four categories of the Needs Near End of Life (NEST): physical symptoms (47 / 50, 94%), finances (36 / 50, 72%), mental health (31 / 50, 62%), and access to care (29 / 50, 58%). The majority of patients reported moderate to severe fatigue, pain, dyspnea, and depression on the ESAS.
Temel et al. 2010	151 patients with metastatic lung cancer	To examine the effect of early palliative care integrated with standard oncologic care on patient- reported outcomes, the use of health services, and the quality of end-of-life care among patients with metastatic non-small-cell lung cancer.	Non-blinded, randomized, controlled trial	Patients assigned to early palliative care had a better quality of life than did patients assigned to standard care (mean score on the FACT-L scale, in which scores range from 0 to 136, with higher scores indicating better quality of life], 98.0 vs. 91.5; P = 0.03). In addition, fewer patients in the palliative care group than in the standard care group had depressive symptoms (16% vs. 38%, *P* = 0.01). Despite the fact that fewer patients in the early palliative care group than in the standard care group received aggressive end-of-life care (33% vs. 54%, P = 0.05), median survival was longer among patients receiving early palliative care (11.6 months vs. 8.9 months, P = 0.02).
Glare, et al. 2011.	119 patients in a GI oncology practice specializing in colorectal cancer and neuroendocrine tumors	To explore the implementation of the NCCN screening and referral criteria in an outpatient GI oncology practice.	Cross-sectional survey	Using the 24 items for NCCN referral criteria to screen for specialist palliative care provider, identified 7 to 17% of patients as having PC issues and 13% of patients who might benefit from specialist referral.
Glare, et al. 2013	194 gastrointestinal oncology patients	To evaluate the feasibility and impact of implementing the NCCN Guidelines referral criteria as a trigger for PC consults	Cross-sectional survey	Using the NCCN guidelines as a referral trigger, patients had a significant increase in access to the PC service, and appeared to occur earlier in the course of the disease. Almost two-thirds (73%) of patients would meet the criteria for a PC consult.
Hui et al. 2014	366 cancer patients with PC referral and quality of care indicators	To examine how the timing and setting of PC referral were associated with the quality of end-of-life care	Retrospective study	Earlier PC referral was associated with fewer emergency room visits (39% vs 68%; P < .001), fewer hospitalizations (48% vs 81%; *P* < .003), and fewer hospital deaths (17% vs 31%; P= .004) in the last 30 days of life. Similarly, outpatient PC referral was associated with fewer emergency room visits (48% vs 68%; *P* < .001), fewer hospital admissions (52% vs 86%; P < .001), fewer hospital deaths (18% vs 34%; P5.001), and fewer intensive care unit admissions (4% vs 14%; P5.001). In multivariate analysis, outpatient PC referral (odds ratio [OR], 0.42; 95% confidence interval [CI], 0.28–0.66; P < .001) was independently associated with less aggressive end-of-life care. Men (OR, 1.63; 95% CI, 1.06- 2.50; P5.03) and hematologic malignancies (OR, 2.57; 95% CI, 1.18–5.59; P5.02) were associated with more aggressive end-of-life care.
Bakitas et al. 2015	207 patients with advanced cancer	To compare the effect of early versus delayed PC on quality of life (QOL), symptom impact, mood, 1- year survival, and resource use.	Randomized controlled trial	Patient-reported outcomes and resource use were not statistically significant between early versus delayed referral to palliative care. However, the 1-year survival rates after enrollment was improved with those in the early group (63%) compared to 48% in the
				delayed group (difference, 15%; P = .038). Relative rates of early to delayed decedents’ resource use were similar for hospital days, intensive care unit days, emergency room visits, chemotherapy in last 14 days, and home death.
Rocque et al. 2015	203 patients with hematologic malignancies	To evaluate the implementation of triggered palliative care consultation (TPCC) as part of standard care	Prospective, pre-post, sequential cohort study	Implementation of TPPC significantly improved patients’ prognostic awareness of their cancer from 65 to 94%, enhanced the communication between the patient, PC provider and was viewed favorably by 74% of the oncologists. TPCC had minimal impact on hospice utilization, cost of care, survival, patient reported symptoms, and patient satisfaction, likely because of the limited nature of the intervention.
Hui et al. 2016	60 international experts on palliative care	To develop consensus on a list of criteria for referral of patients with advanced cancer at secondary or tertiary care hospitals to outpatient palliative care	Delphi method using a structured communication technique to establish a convergence of opinion.	Panelists reached consensus on 11 major and 36 minor criteria for referral to palliative care (11 major criteria: severe physical symptoms, severe emotional symptoms, request for hastened death, spiritual or existential crisis, assistance with decision making or care planning, patient request for referral, delirium, spinal cord compression, brain or leptomeningeal metastases, within 3 months of advanced cancer diagnosis for patients with median survival of 1 year or less, and progressive disease despite second-line therapy. Consensus was also reached on 36 minor criteria for specialist palliative- care referral.
Adelson et al. 2017.	113 inpatients with solid tumors	To develop and test four standardized criteria for automatic PC consultation on the inpatient solid tumor service.	Prospective cohort study	Automatic PC consultation using a standardized criteria decreased the 30-day readmission rates from 35 to 18% (P = .04), hospice referral rates increased from 14 to 26% (P = .03), and receipt of chemotherapy post-discharge decreased from 44 to 18% (P = .03). There was no significant change in LOS (*P* = .15) or use of
				the ICU (*P* = .11) between the groups. Patients in the intervention group were more likely to be discharged to home with any home-based services
Molin, et al.	840	To explore the use	Prospective	The PALLIA-10 questionnaire
2019	hospitalized	of the PALLIA-10	multicenter	score appeared to be a reliable
	adult patients	questionnaire in	study	predictive factor to refer patients
	in conventional	advanced cancer		to PC team intervention, and
	medicine or in	patients		prognostic factor for patients
	radiotherapy			scored 4–5 and > 5. In addition, the
	departments			PALLIA-10 score appeared as a
				reliable prognostic factor for
				death at 6 months, independent
				from the variation of other
				severity criteria.
Brinkman-	535	To investigate the	Prospective,	No significant difference in
Stoppelenburg	hospitalized	association	observational	hospital costs between patients
et al. 2019	patients with	between	study	with PCT as compared to patients
	incurable	palliative care team		without PCT consultation. Patients
	cancer	(PCT) consultation		with PCT consult had a worse life
		and the content and		expectancy, performance status
		costs of hospital		and more often had no more
		care		options for anti-tumor therapy.
				Hospital length of stay, use of
				most diagnostic procedures,
				medication and other therapeutic
				interventions were similar.
Hui et al.	200 patients	To examine the	Retrospective	Among the outpatient palliative
2020	with advanced	proportion of	study	care referral, the median overall
	cancer	patients referred to		survival from was 14 (95%
		the PC clinic who		confidence interval 9.2, 17.5)
		met the		months. A majority (*n* = 170, 85%)
		standardized		of patients met at least 1 major
		criteria and its		criteria; specifically, 28, 30%,
		timing for referral		20, and 8% met 1, 2, 3, and ≥ 4
		to the MDACC		criteria, respectively. The most
		Supportive Care		commonly met need-based
		Outpatient Clinic		criteria were severe physical
				symptoms (*n* = 140, 70%),
				emotional symptoms (*n* = 36,
				18%), decision-making needs (n =
				26, 13%), and
				brain/leptomeningeal metastases
				(*n* = 25, 13%). For time-based
				criteria, 54 (27%) were referred
				within 3 months of diagnosis of
				advanced cancer and 63 (32%)
				after progression from ≥2 lines of
				palliative systemic therapy. The
				median duration from patient first
				meeting any criterion to palliative
				care referral was 2.4 (interquartile range 0.1, 8.6) months
Caraceni, A. et	229 patients	To identify timing	Observational	Referral to Palliative care
al. (2020)	with thoracic	and factors	retrospective	Outpatient Clinic (POC) was
	malignancies	associated to PC	study	significantly higher for patients
		referral in patients		with worse performance status
		with thoracic		(PS) (HR = 4.5), more advanced
		malignancies, and		disease stage (HR = 3.1), pain
		to describe their		(HR = 4.9), dyspnea (HR = 2.5) and
		clinical care		cough
		pathway.		(HR = 2.2). The multivariable model
				confirmed independent
				prognostic value for PS, disease
				stage and pain. Results suggest
				considering symptom burden, PS
				and disease stage as screening
				criteria for referral to PC in
				patients with thoracic
				malignancies.
Gemmel, R. et	159 patients	To identify the	Retrospective	Of the 159 patients identified, 46
al	who died	prevalence of	cohort study	% were referred to palliative care
(2020)	during hospital	cancer		prior to terminal admission.
	admission, who	patients who died		Application of 6 out of 7 trigger
	met criteria for	during a non-		tools would have resulted in the
	palliative care	elective hospital		majority of patients (up to 91.2%)
	consultation	admission, who met		referred to palliative care prior to
		the criteria for a		admission. Most patients (52.2%)
		palliative care		were referred only during their
		consultation within		terminal admission. Patients
		the 6 months prior		known to palliative care before
		to death according		admission (*N* = 73) were reviewed
		to a number of		quicker than those who were not
		palliative care		(*N* = 86) (median (range)1 day (0–23
		referral trigger		days) versus 5 days (0–59 days),
		tools.		*p* < 0.00001).
Hansen, MB et	31,139 adult	To investigate if the	Retrospective	Clinically neglectable associations
al (2020)	cancer patients	symptomatology	review	were found between patients
	registered in	(EORTC QLQ-C15-		referred by the general
	the Danish	PAL questionnaire)		practitioner and hospital
	Palliative Care	differed for patients		physician related to symptoms
	database	referred to		(pain, appetite loss, fatigue),
		specialized palliative		number of symptoms/problems,
		care from general		number of severe symptoms/
		practitioners in the		problems (odds ratios between
		primary healthcare		1.05 and 1.20, all *p* < 0.05) and
		sector and for		physical functioning (odds ratio=
		patients referred by		0.81 (inpatient care) and 1.32
		hospital physicians		(outpatient), both *p* < 0.05). The
		in the secondary		survival time from referral to
		healthcare sector.		specialized palliative care was on
				average longer for patients
				included in the study. The mean
				number of symptoms/ problems were very similar for patients referred by the general practitioner and hospital physicians. The difference between patients referred by the general practitioner and the hospital physician did not seem to be clinically relevant for any of the symptoms/problems or overall QOL.

Table 3).

### Referral criteria

Four studies [26, 30, 33, 34] identified and characterized the PC needs of cancer patients using questionnaires and assessment instruments. Sanders et al. (2010) reported that patients’ greatest PC needs were in the physical and daily living domain, followed by psychological needs, health system and informational needs, and patient care support needs. The most common unmet need was a lack of energy and tiredness (75%). Grudzen et al. (2010) used the validated assessment instruments Needs at the End- of-Life Screening Tool (NEST) [39], McGill Quality of Life Questionnaire (MQOL) [40],and Edmonton Symptom Assessment System (ESAS) [41] to assess (1) the range and severity of symptoms, (2) goals of care, (3) psychological well-being, (4) health care utilization, (5) spirituality, (6) social connectedness, (7) financial burden, (8) the patient–clinician relationship, and (9) overall quality of life. Results showed that in all nine of the above categories, more than 50% of patients suffered not just from physical symptoms (47/50, 94%), but also from mental distress (31/50, 62%), financial hardship (36/50, 72%), mental health (31/50, 62%, and difficulty accessing care (29/50, 58%). The majority of patients reported moderate to severe fatigue, pain, dyspnea, and depression on the ESAS [41]. Caraceni et al. (2020) used multidimensional systematic symptom assessment to determine referral to PC, hospice, or home care when needed. Patients with the highest symptom burden were more likely to be referred earlier, and 75% of them died within 1 year from referral. Among symptoms, frequent reasons for referral included pain, respiratory symptoms, asthenia, and loss of appetite. Other clinical conditions associated with referral were deterioration of performance status and presence of brain metastases.

Molin et al. (2019) explored the use of the PALLIA-10 questionnaire for referral of advanced cancer patients to a dedicated PC team. PALLIA-10 is a multidimensional 10-item screening form addressing medical, psycho-social, and ethical issues with scoring from 0 to 10 to categorize patients by their PC requirement. Results showed that patients were significantly more frequently referred to a PC team when their PALLIA-10 score was >3 (adjusted odds ratio, 2.6; 95% CI, 1.65-4.11). PALLIA-10 score appeared to be a reliable and prognostic instrument for identifying patients for PC referral.

To facilitate PC referral, a panel of 60 international palliative care experts developed a list of criteria for referral of patients with advanced cancer for outpatient palliative care [42]. Using the Delphi study methodology, the panelists rated 39 needs-based criteria and 22 time-based criteria. Of those, they reached consensus on 11 major criteria for referral which includes: severe physical symptoms, severe emotional symptoms, request for hastened death, spiritual or existential crisis, assistance with decision making or care planning, patient request for referral, delirium, spinal cord compression, brain or leptomeningeal metastases, within 3 months of advanced cancer diagnosis for patients with median survival of 1 year or less, and progressive disease despite second-line therapy. Consensus was also reached on 36 minor criteria for specialist palliative-care referral.

### Outcomes

Of the eight studies that explored the outcomes of PC in patients with cancer, two randomized controlled trials [28, 38] evaluated the benefit of early versus delayed PC referral on patient reported outcomes including quality of life (QOL), symptom impact, mood, survival and resource use. In the study of 151 patients with newly diagnosed metastatic non-small cell lung cancer, patients assigned to early PC had a better quality of life than did patients assigned to standard care [28]. In addition, even though there were fewer patients in the early PC group than in the standard care group that received aggressive end-of-life care (33% vs. 54%, P = 0.05), median survival was longer among patients receiving early palliative care (11.6 months vs. 8.9 months, P = 0.02). Conversely, in another study comparing the effect of early versus delayed PC referral in 207 patients with advanced cancer, patient-reported outcomes (quality of life, symptom impact, mood, 1-year survival), and resource use were not significantly different between patients with early referral than those with delayed referral [38]. However, the 1-year survival rate after study enrollment was improved in the early group (63%) as compared with the delayed group (48%; P = 0.038). Relative rates of resource use in early and delayed decedents were similar for hospital days (0.73; 95% CI, 0.41 to 1.27; p=.26), intensive care unit days (0.68; 95% CI, 0.23 to 2.02; p= .49), emergency room visits (0.73; 95% CI, 0.45 to 1.19; *p* = .21), chemotherapy in last 14 days (1.57; 95% CI, 0.37 to 6.7; *p* = .27, and home death (27 [54%] v 28 [47%];*p* = .60).

Three studies [24, 25, 29] evaluated the implementation of standardized criteria or triggers for palliative care (PC) consultation on the inpatient service for patients with solid tumors, advanced cancer, and its impact on the quality of cancer care. In patients with solid tumors, when standardized criteria for PC consultation were used, PC consultations doubled from 19 of 48 (39%) to 52 of 65 (80%), P < .001; 30-day readmissions declined from 17 of 48 (35%) to 13 of 65 (18%), P = .04; hospice referrals increased from seven of 48 (14%) to 17 of 65 (26%), P = .03; and receipt of chemotherapy after discharge decreased from 21 of 48 (44%) to 12 of 65 (18%), P = .03 [25]. In patients with advanced cancer, Rocque et al. (2015) also noted that triggered PC consultation significantly improved patients’ prognostic awareness of their cancer from 65% to 94%, enhanced the communication between the patient and PC provider, and was viewed favorably by 74% of the oncologists [24]. Similarly, using the NCCN guidelines’ criteria as a trigger for PC referral resulted in a significant increase in patients’ access to PC service, and PC referral also appeared to occur earlier in the course of the disease as a result [35, 36]. However, triggered PC consultation had minimal impact on hospice utilization, cost of care, survival, patient-reported symptoms, and patient satisfaction [23]. In addition, using the standardized criteria for automatic PC consultation did not significantly change length of stay (P = 0.15) or use of the intensive care unit (P = 0.11) [25], or hospital costs [27].

### Heart failure

A total of 292,699 patients were included in the 14 studies of HF and PC (Table 4). Eight studies were retrospective, and six were prospective, with two of the included studies evaluating different data from the same randomized controlled trial of PC intervention. Eight studies looked at referral criteria only, five studies evaluated outcomes only, and one study evaluated both referral criteria and outcomes.

**Table 4:** Palliative/Supportive Care in Patients with Heart Failure

Study	Population	Aims	Design	Key Findings
Harding et al. (2009)	365 adult HF inpatients in tertiary teaching hospitals in the UK	1) To measure point prevalence of inpatients appropriate for PC 2) To identify patient characteristics associated with PC appropriateness to inform referral criteria 3) To propose evidence-based clinical referral criteria	Cross-sectional design, identifying chronic HF as a reason for current admission, using NYHA stage 3/4 classification, cross- referenced with existing echocardiogram data	Proposed criteria for PC referral for patients with chronic HF: 1. Symptomatic (e.g. breathless at rest or on minimal exertion) despite optimal treatment 2. On optimal therapy but with continuing or deteriorating physical or psychological symptoms 3. HF patients when hospital admission may not be the best/only/preferred option, or for whom PC (hospice, day care, hospital inpatient or community care) may be of benefit, either immediately or in the future 4. Where the family or carer(s) would benefit from support, either immediately or in the future (including bereavement) 5. Where patient has had 2 or more previous admissions for HFwithin the last 6 months
James et al. (2010)	214 patients with a discharge diagnosis of HF	To determine if SHFM can identify HF inpatients who would benefit from PC referrals	Cohort, retrospective and prospective Medical records	The SHFM13 is a Web-based tool that uses specific clinical and laboratory variables, HF medications, and devices the patient currently has or will receive as predictor variables. Clinical variables entered into the tool include age, sex, NYHA classification, ejection fraction, ischemic cardiomyopathy, QRS duration, systolic blood pressure, and devices such as pacemakers and intraventricular conduction devices. 63% of HF patients with life expectancy ≤1.5 years would have received timely PC consultation had the SHFM been used as a screening tool.
Ng Fat Hing et al. (2018)	612 patients with advanced NYHA HF and left ventricular ejection fraction ≤40%	To use the SHFM as a prediction of 1- year outcomes to help inform decision-making	Retrospective, chart review	SHFM showed good discrimination for outcomes including 1-year event-free survival from death, heart transplant, and ventricular assist device implant among low- to moderate-risk patients but underestimated events in high-risk patients.
Avula et al. (2020)	689 patients with HF	To evaluate the SHFM and PRISM score to predict 1- year mortality	Retrospective	The discriminatory ability of modified SHFM was similar to that of the PRISM score, but the models in combination significantly improved the ability to predict 1-year mortality (*P* = 0.002).
Ezekowitz et al. (2011)	105 patients (mean age = 65 years, 76% male, mean ejection fraction = 28%) followed up in outpatient HF clinics	To assess the utility of PC questionnaires (NYHA, PPS, ESAS, and KCCQ) in patients with HF	Cohort, prospective	The PPS and ESAS were each correlated to the NYHA class (P < 0.0001 for both) and the KCCQ score (PPS: R2 = 0.57; ESAS: R2 = −0.72; both *P* < 0.0001). 33 patients died (10 patients) or were hospitalized (26 patients) for more than 1 year. In addition to age and sex, a higher (worse) ESAS score trended toward significance (P = 0.07) and a lower (worse) PPS was significant (P = 0.04) in predicting all-cause hospitalization or death. Given the difficulty of identifying patients with HF eligible for PC or hospice care, these tools may be of use in clinical practice.
Greener et al. (2014)	2647 patients with HF admissions who received and did not receive PC services	To identify individual-level predictors of palliative care referral for HF patients	Chart review, retrospective	6.2% of HF patients were referred to PC during their hospitalization. Patients who were referred to PC were older (> 75 years), more likely to be married, and had longer hospital stays (19.53 days versus 9.67 days; P < 0.0001), higher risk for mortality (score of 3.31 versus 2.56; P < 0.0001), higher severity of illness (score of 3.30 versus 2.85; P < 0.0001), more days in the intensive care unit (4.96 days versus 2.01 days; *P* = 0.03), more prior-year HF admissions (*P* = 0.0004), and more hospital readmissions within 30 days (P < 0.0001). PC-referred patients were also more likely to have chronic and acute renal failure and Alzheimer disease, to be deceased at discharge or to be discharged to hospice care, and to undergo thoracentesis.
Campbell et al. (2018)	272 patients screened for specialized PC needs	To develop a definition of specialized PC needs and assess outcomes of those	Prospective, observational	27% of patients had specialized PC needs, and these patients were older (*P* = 0.041); had lower SBP (P = 0.018), more severe NYHA class (*P* = 0.031), lower scores on AKPS
		who received specialized PC		and NAT-PD-HF (*P* < 0.001 and 0.008), and higher Zarit Burden Interview severity (*P* < 0.001); and were more likely to have a history of myocardial infarction (*P* = 0.004) and a history of diabetes (P = 0.029).
Kane et al. (2018)	372 patients screened for recruitment into PC intervention	To identify patients for recruitment into PC interventions using modified European Society of Cardiology and NYHA inclusion criteria	Prospective, observational	NYHA II patients have PC needs and limiting referral to PC to only NYHA III/IV is not recommended. Including NYHA II patients will improve recruitment to PC treatment plans.
Roch et al. (2020)	100 patients hospitalized with HF	To evaluate an integrated PC outcome scale for assessing PC needs in patients with HF	Cross-sectional study	The integrated PC outcome scale identified clinically relevant somatic and psycho-emotional symptoms in approximately 75% of patients. Patients also found the assessment to be easy to understand (95%) and felt it was a suitable tool to assess PC needs (91%).
Unroe et al.	229,543	To examine	Retrospective	Approximately 80% of Medicare
(2011)	Medicare	resource use in the	cohort study	beneficiary patients were
	beneficiaries	last 180 days of life,		hospitalized in the last 6 months
	with HF who died	including all-cause		of life; days in intensive care
	between	hospitalizations,		increased from 3.5 to 4.6 days (P <
	January 1, 2000,	intensive care unit		0.001). Use of hospice increased
	and December	days, skilled		from 19% to nearly 40% of
	31, 2007	nursing facility		patients (P < 0.001). Unadjusted
		stays, home health,		mean costs to Medicare per
		hospice, durable		patient rose 26% from $28,766 to
		medical		$36,216 (P < 0.001). After
		equipment,		adjustment for age, sex, race,
		outpatient		comorbid conditions, and
		physician visits, and		geographic region, costs increased
		cardiac procedures.		by 11% (cost ratio, 1.11; 95% CI,
				1.10–1.13). Increasing age was
				strongly and independently
				associated with lower costs. Renal
				disease, chronic obstructive
				pulmonary disease, and black race
				were independent predictors of
				higher costs.
Kheirbek et	179 hospice-	To examine the	Chart review,	30-day all-cause readmission rate
al. (2015)	referred patients	association of	retrospective	was 5% in the hospice-referred
	matched with	discharge hospice		group and 41% in the hospice-
	179 hospice-	referral with 30 day		eligible group, corresponding to
	eligible patients	all cause		an HR of 0.12 (95% CI, 0.06–0.24)
				for hospice referral. Hospice-
		readmission in decompensated HF		referred patients were admitted later. 30-day mortality was higher in the hospice-referred group (43% versus 27%) with an HR of 1.86 (95% CI, 1.30–2.67). However, among patients who were alive at 30 days, all-cause readmission occurred in 8% of the hospice- referred group versus 39% of the hospice-eligible group (HR = 0.17; 95% CI, 0.08–0.36).
Rogers et al. (2017)	150 patients randomized to usual care versus PC intervention	To assess for quality-of-life outcomes in patients receiving usual care versus usual care and PC intervention	Prospective, randomized	Patients with PCintervention had significant improvements in KCCQ and FACIT-Pal scores at 6 months (KCCQ difference: 9.49 points; 95% CI, 0.94–18.05; P = 0.030; FACIT-Pal difference: 11.77 points; 95% CI, 0.84–22.71; P = 0.035). Depression also improved in the PC intervention group (HADS- depression difference: −1.83; *P* = 0.048). Randomization did not affect re-hospitalization or mortality.
Truby et al. (2020)	150 patients with HF	Secondary analysis of trial by Rogers et al. to compare quality of life between men and women	Randomized controlled trial, alternative outcome analysis	Women had lower KCCQ scores (24.5 versus 36.2, P = 0.04), but there was no significant difference in the FACIT-Pal scale (115.7 versus 120.3, *P* = 0.27). After referral to PC, men had significant improvement in KCCQ scores at 6 months, whereas women did not (*P* = 0.047 versus *P* = 0.39).
Liu et al. (2020)	57,272 patients with primary hospital encounter diagnosis of HF or cancer receiving PC consultation	To evaluate outcomes of PC consultations for hospitalized patients with HF and cancer	Retrospective, Palliative Care Quality Network data set (nationwide collaborative of interdisciplinary PC teams)	Patients with HF were older (75.3 versus 65.2 years), had lower Palliative Performance Scale scores (35.6% versus 42.4%), and were more likely to be in a critical care unit (35.3% versus 12.5%) or telemetry or step-down unit (35.2% versus 19.2%) compared with patients with cancer. Patients with HF had more improvement in symptoms of dyspnea (odds ratio, 2.17) after PC referral compared with patients with cancer.

*HF* heart failure; *PC* palliative care; *NYHA* New York Heart Association class; *SHFM* Seattle Heart Failure Model; *PRISM* Placement Resource Indicator for Systems Management; *PPS* Palliative Performance Scale; *ESAS* Edmonton Symptom Assessment System; *KCCQ* Kansas City Cardiomyopathy Questionnaire; *NAT-PD-HF* Needs Assessment Tool–Progressive Disease–Heart Failure; *HR* hazard ratio; *FACIT-Pal* Functional Assessment of Chronic Illness Therapy–Palliative Care; *HADS* Hospital Anxiety and Depression Scale; *AKPS* Australia-modified Karnofsky Performance Status

### Referral criteria

The nine studies that assessed potential referral criteria for PC in patients with HF looked at various factors. Harding et al. (2009) compared characteristics of 28 admitted patients with HF between those who were appropriate for PC referral and those who were not. Patients with HF appropriate for PC referral had more previous admissions, had more multi-professional inpatient staff evaluating them, and were more likely to have a do-not-resuscitate order [43]. Other studies examined the accuracy of tools for predicting survival in HF patients, which could help guide goals-of-care discussion and PC referral. James et al. (2010) retrospectively applied the Seattle Heart Failure Model (SHFM) [44], which predicts lifespan using clinical, medication, laboratory, and intervention data, in patients admitted with HF to evaluate the accuracy of this model and its potential to identify patients who would benefit most from PC referral. The authors concluded that post-intervention SHFM scores could help identify patients for PC referral [45]. In another study, Ng Fat Hing et al. (2018) evaluated the use of SHFM to predict survival and guide when referral to PC should take place [46]. The authors found that SHFM captured the majority of patients who would have died within 1 year (95.3%). However, the SHFM underestimated survival in the highest-risk patients, resulting in only 27% of this patient group being referred at an appropriate time. The study concluded that since the SHFM underestimates survival, many patients would be referred to PC too early, resulting in PC resources being expended unnecessarily. Avula et al. (2020) lso used the SHFM to predict mortality; in addition, the authors used the Placement Resource Indicator for Systems Management (PRISM) score, which is not specific to a disease [47]. The use of PRISM and a modified SHFM in combination significantly improved the ability to predict 1-year mortality in HF patients compared with either model used alone.

Ezekowitz et al. (2011) prospectively evaluated 105 patients in outpatient HF clinics with two validated PC questionnaires (ESAS [41] and Palliative Performance Scale [48]) and two validated HF assessments (NYHA functional class [5] and Kansas City Cardiomyopathy Questionnaire [KCCQ] [49, 50]) and found significant correlation between the PC and HF assessments (P < 0.0001 for each PC assessment compared with NYHA class and compared with the KCCQ). The authors proposed that since the ESAS and Palliative Performance Scale showed good correlation with traditional HF scores, they could be useful in assessing HF patients for PC referral [51]. In a retrospective single-center study of all patients admitted for HF between 2005 and 2010, Greener et al. (2014) found that 6.2% were referred to PC, and multivariable logistic regression analysis found several predictors of PC referral, including previous HF-related hospitalizations, admission to the intensive care unit, older age, married status, and higher severity of illness. The authors speculated that being married was a predictor for PC referral because PC services provide resources not only for the patient but also for family members and caretakers [52]. Campbell et al. (2018) performed a prospective observational study of 272 patients and found that those needing a PC specialist were more likely to have been hospitalized for HF in the preceding 6 months and had a worse NYHA class, lower KCCQ score, and worse performance status assessed by a physician (Australia-modified Karnofsky Performance Status) [50]. However, in contrast to the previous studies presented, patients needing a PC specialist were younger (P = 0.076) and did not differ in number of comorbidities [53].

Kane et al. (2018) evaluated recruitment strategies for 372 patients with HF to undergo a PC needs assessment and ultimately compared 25 patients who completed the PC intervention. The authors found that using NYHA class as a criterion for referral to PC was problematic for two main reasons: (1) NYHA class can change owing to changes in volume status, so a proportion of patients with NYHA class II may have PC needs but momentarily appear too healthy for PC. (2) Assessment of NYHA class can be subjective, as seen in differences in application of NYHA class between sites included in the study. The authors recommended using indicators other than NYHA class for PC referral, as done in the 2016 European Society of Cardiology definition of HF [54]. Finally, Roch et al. (2020) evaluated an integrated PC outcome scale, which identified relevant symptoms for PC referral in 75% of patients and was determined by a vast majority of patients (95%) to be an easy tool to understand. The study highlighted the importance of using tools accepted by both the patients and the providers when assessing PC referral [55].

### Outcomes

A total of 287,595 patients were included in the six studies evaluating outcomes after PC referral. In a retrospective study of resource use near the end of life among 229,543 Medicare beneficiaries with HF, Unroe et al. (2011) found that hospice referral increased from 19% to almost 40% from 2000 to 2007 however, costs remained elevated, and use of other services such as inpatient hospitalization and echocardiograms did not decrease. Many patients had short hospice stays, with 37% having stays less than 7 days, and the authors surmised that this short duration may have prevented patients and families from receiving the full benefit of hospice services, owing to the late referral [56]. Kheirbek et al. (2015) matched 179 hospice-referred patients with propensity-matched hospice-eligible patients and found that readmission rates were lower for the hospice-referred patients up to 6 months after discharge, including 30-day readmission. However, one possible explanation for the lower re-admission rate was the fact that over 40% of patients in the hospice-referred group died in the first 30 days after discharge, suggesting, again as the aforementioned study also stated, that PC referral occurred too late [57].

In the only randomized trial of PC intervention in HF patients, the primary endpoint for 150 patients was quality-of-life change at 6 months as assessed by the KCCQ and Functional Assessment of Chronic Illness Therapy–Palliative Care scale (FACIT-Pal) [58]. Patients with PC referral had statistically significant improvements in quality of life (KCCQ: P = 0.030, FACIT-Pal: P = 0.035) compared with those with usual care; however, mortality was not affected and, as in other studies in our analysis, re-hospitalization was not affected [59]. A secondary analysis of the same trial evaluated differences in quality of life between men and women and found that men had significant improvement in KCCQ scores at 6 months, whereas women did not (P = 0.047 versus P = 0.39). Campbell et al. (2018), in addition to assessing predictors of needing specialist PC, also prospectively evaluated outcomes of patients with HF. Only 24% of patients meeting criteria for needing specialist PC actually received PC. The patients meeting these criteria had significantly fewer days alive out of the hospital (P < 0.001) compared with patients not meeting the criteria, but declines in quality of life were similar between groups, as assessed by ESAS and KCCQ scores [53].

## Discussion

Cardio-oncology is already a multidisciplinary specialty with unique considerations for patient care when cancer coexists with cardiovascular disease, including HF. The added complexities of PC needs make this patient group a complicated one to treat. Many studies have evaluated PC in patients with cancer or HF, but a dearth of evidence exists regarding patients with both. This systematic review reveals areas of overlap and potential improvement for identifying PC referral criteria and assessing outcomes of PC intervention in these patients.

Cancer and HF share similarities in their patient-reported symptoms, quality of life, symptom burden, social support needs, readmission rates, and mortality. Symptoms evaluated in questionnaires such as the ESAS for cancer and the KCCQ for HF commonly include fatigue and dyspnea assessments. This overlap may be a reason for the significant correlation found by Ezekowitz et al. (2011) between the ESAS and KCCQ assessments (P < 0.0001). In addition to quantifying the severity of these symptoms, the questionnaires also quantify the degree to which the patient’s quality of life is affected by the symptoms. Importantly, ESAS and KCCQ are patient-reported symptoms, as opposed to provider assessments, and patients are often more concerned with how they feel than with etiology or pathogenesis. Providers from both oncology and cardiology can address patient symptoms with PC intervention, regardless of whether symptoms are due to cancer or to HF. This multidisciplinary relevance is the strength of using patient- reported outcomes and there has been an increase in studies validating their use for various cancer and HF subtypes. Other symptoms that have been identified in both cancer and HF include anxiety, distress, delirium, and depression. Future studies of PC in patients with both cancer and HF should include quantification of all of these symptoms and their impact on quality of life.

One often-overlooked strength of PC intervention is the social support for caregivers in addition to the patient. The assessment of caregiver needs was addressed more in the included studies of cancer, while only one of the HF studies assessed this need. This difference may be due to the higher number of citations for PC with cancer compared to citations for PC with HF. Another possible explanation is the understanding and impression of a cancer diagnosis on patients and their families, compared with that of a diagnosis of HF. Many patients and family members associate cancer with a high risk of death; however, HF has worse mortality than many cancers but does not have the same stigma of death. Another factor is the predictability and duration of cancer treatment. Family members can plan for expected declines after chemotherapy or radiation and allocate the time needed to care for the patient. Therefore, increased use of caregiver-needs assessments are needed in the evaluation of patients with HF for PC.

With the increasing incidence of cardiotoxicity from anticancer agents that can result in HF, a collaboration between oncology and cardiology is paramount for the integration of PC to manage the complex issues in cardio-oncology patients. Although early referral to PC has been shown to improve outcomes, yet, PC referrals remain delayed because of a lack of criteria on who should be referred or the optimal timing for referral. To facilitate the incorporation of PC in the care of patients with cancer and HF, a set of criteria that address both cancer and HF are necessary. Table 5 shows a list of the referral criteria important for PC intervention in patients with cancer and patients with HF and identifies areas where there is overlap between cancer and HF. The next step would be to conduct a Delphi study on a combination of these proposed criteria to develop a consensus among the cardio-oncology experts on a list of criteria for PC referral for patients with cancer and HF. These criteria, if validated, could provide guidance for identification of patients suitable for referral to PC, and could help streamline and standardize clinical practice, research and health care resources for this increasing number of patients.

**Table 5:** Proposed Referral Criteria for PC for Patients with Cancer and Heart Failure

Criteria for referral		Used in patients with	
		**Cancer**	**Heart** **failure**
Need-based criteria	Anxiety (severe)	(H)	Ng
	Assistance with decision making or care planning	(H)	
	Brain or leptomeningeal metastases	(H, C)	
	Caregiving needs (family, caregiver limitations)	(G, GL, N, S)	Ha
	Cognitive impairment	(GL)	
	Communication barriers (language, physical)	(GL)	
	Deteriorating symptoms even with optimal therapy		(Ha)
	Do-not-resuscitate order		Ha
	Dyspnea (severe)	A, G	E
	Fatigue (severe)	(S, G)	(Ng)
	Financial hardship	(S, G, GL)	
	Health system and informational needs	(S)	
	History of drug or alcohol abuse	(G, GL)	
	Inadequate social support	(G)	
	Moderate to severe distress, delirium, depression	(A, H, G, GL, N, S)	(Gr, Ng)
	Multiple adverse reactions to pain and symptom management interventions	(G)	
	Older age (> 75 years)		(Gr)
	Pain (including neuropathic)	(A, G, GL, S, C)	
	Patient request for referral	(N,H)	
	Psychological distress/needs	(GL, S)	
	Psychiatric disorder	(G)	
	Rapid escalation of opioid dose	(GL)	
	Request for hastened death	(G, GL, H)	
	Severe physical symptoms	(A,G, H, S, N, C)	Ha
	Secondary diagnosis of Alzheimer disease	(Gr)	
	Spiritual or existential crisis	(G, S, H)	
	Thoracentesis (multiple and recurrent episodes)		Gr
Time-based criteria	3 months of advanced cancer diagnosis for patients with a median survival of 1 year or less (≤1.5 years expected life span)	(A, H)	(J)
	Higher severity of illness		(Gr)
	Limited treatment options, especially in patients receiving phase I therapy or anticancer therapy with a palliative intent	(G)	(Ng)
Illness trajectory criteria	Health care utilization (> 2 hospital admissions within the last 6 months)	(S, A)	(Gr, Ha)
	Low KCCQ score		(Gr)
	Poor prognosis despite second-line therapy	(H, N)	
	Serious comorbid conditions (acute renal failure)	(GL, N)	(Gr)
Worse performance status (NYHA IV)	(Gr, Ha)		

Adelson = A, C = Caraceni, E = Ezekowitz, Glare = GL, Greener = Gr, Grudzen = G, Ha = Harding, H = Hui, J

= James, N = NCCN, Ng = Ng Fat Hing, S = Sanders

Common outcome measures between PC intervention for HF and cancer include quality-of-life assessments through patient-reported outcomes, readmission rates, and mortality. As observed in the use of symptom and quality-of-life questionnaires for referral to PC, there is overlap between cancer and HF patients in the use of these assessments as outcomes. Also, in both HF and cancer, readmission rates and mortality are used as significant outcomes to evaluate the efficacy of PC intervention. Mortality is a difficult measure for assessing PC efficacy, as aggressive life-saving measures will often not be pursued after PC referral, a choice that may contribute to early mortality. Furthermore, as highlighted in studies from this review, if PC referral occurs too late, the full benefit of PC services is not realized, as patients in one study passed away for an average of 7 days after referral. The outcomes of PC are thus tied with the timing of referral. Furthermore, the symptom assessment tools have limitations in their prediction of lifespan often overestimating or underestimating this important factor. Therefore, part of optimizing outcomes is assessing the time-based criteria for referral noted in Table 5. Further research is needed to improve individual lifespan prediction after a diagnosis of cancer and HF, which will then improve outcomes by enabling appropriate timing of PC referral.

**Fig. 2:**
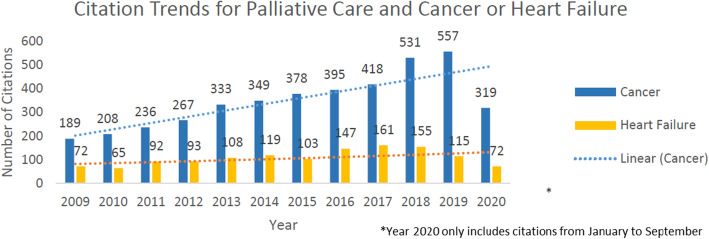
Palliative care citations for cancer and heart failure

Increasing awareness and recognition of PC as an important consideration for patients with cancer and HF is evident from the increased number of citations on this topic every year (Figure 2). Still, cancer citations outnumber those of HF 2 to 1, and it is clear that more progress is needed to improve utilization of PC in patients with HF. At the time of this review, there is only one randomized controlled trial comparing the use of PC versus usual care in patients with HF. Even more understudied is the niche field of cardio-oncology involving PC referral in patients with both cancer and HF. More studies are needed to better delineate PC in this specialized patient population.

### Limitations

The searches were limited to English only. Inclusion of articles in languages other than English may have broadened our results, but translation of these articles was not feasible. The limitations of this review were the lack of a quantitative statistical meta-analytic comparison of studies because of the heterogeneity of the study designs, participants, interventions, and reported outcome measures.

## Conclusion

The complexities of the multiple issues confronting patients diagnosed with cancer and concurrent HF present challenges in decision-making regarding PC initiation. The multiple comorbidities of this population and the unpredictable illness trajectory of HF add to the complexity of prognostication, particularly with the potential for sudden cardiac death. This systematic literature review provides evidenced-based data to inform the development of criteria for PC referral for patients with cancer and concurrent HF, being mindful that referrals should not rely only on end-of-life or terminal stages. Integrating PC in cardio-oncology, particularly in the management of HF in patients with cancer, as early as at diagnosis, will enable patients, family members, and healthcare professionals to make informed decisions about various treatments and end-of-life care and provide an opportunity for patients to participate in the decisions about when and where they will spend their final days. Additional research is needed to develop and validate clinically useful criteria for PC referral to prospectively identify cancer patients with a concurrent diagnosis of HF patients who may most benefit from PC referral.

## Abbreviations

ESAS: Edmonton Symptom Assessment System
FACIT-Pal: Functional Assessment of Chronic Illness Therapy–Palliative Care
HF: heart failure
KCCQ: Kansas City Cardiomyopathy Questionnaire
NCCN: National Comprehensive Cancer Network
NYHA: New York Heart Association
PC: palliative care
SHFM: Seattle Heart Failure Model

## References

[1] Xu J (2020). Mortality in the United States, 2018. NCHS Data Brief.

[2] World Health Organization (WHO). WHO definition of palliative care 2002; Available from: http://www.who.int/cancer/palliateive/definition/en/.

[3] National Institute of Aging. [cited 2021 July 28, 2021]; Available from: https://www.nia.nih.gov/health/what-are-palliative-care-and-hospice-care.

[4] Lynn J, Teno JM, Phillips RS, Wu AW, Desbiens N, Harrold J (1997). Perceptions by family members of the dying experience of older and seriously ill patients. SUPPORT investigators. Study to understand prognoses and preferences for outcomes and risks of treatments. Ann Intern Med..

[5] *The Criteria Committee of the New York Heart Association. Nomenclature and Criteria for Diagnosis of Diseases of the Heart and Great Vessels.* 9th ed. ed. 1994: Little, Brown & Co.

[6] Blinderman CD, Homel P, Billings JA, Portenoy RK, Tennstedt SL (2008). Symptom distress and quality of life in patients with advanced congestive heart failure. J Pain Symptom Manag..

[7] Bekelman DB, Rumsfeld JS, Havranek EP, Yamashita TE, Hutt E, Gottlieb SH (2009). Symptom burden, depression, and spiritual well-being: a comparison of heart failure and advanced cancer patients. J Gen Intern Med..

[8] Bekelman DB, Havranek EP, Becker DM, Kutner JS, Peterson PN, Wittstein IS (2007). Symptoms, depression, and quality of life in patients with heart failure. J Card Fail..

[9] Gadoud A, Kane E, Macleod U, Ansell P, Oliver S, Johnson M (2014). Palliative care among heart failure patients in primary care: a comparison to cancer patients using English family practice data. PLoS One..

[10] Setoguchi S, Glynn RJ, Stedman M, Flavell CM, Levin R, Stevenson LW (2010). Hospice, opiates, and acute care service use among the elderly before death from heart failure or cancer. Am Heart J..

[11] Bakitas M, MacMartin M, Trzepkowski K, Robert A, Jackson L, Brown JR (2013). Palliative care consultations for heart failure patients: how many, when, and why?. J Card Fail..

[12] Institute of Medicine (2015). Dying in America: Improving Quality and Honoring Individual Preferences Near the End of Life.

[13] Tulsky JA (2015). Improving quality of care for serious illness: findings and recommendations of the Institute of Medicine report on dying in America. JAMA Intern Med..

[14] Lindenfeld J (2010). HFSA 2010 comprehensive heart failure practice guideline. J Card Fail..

[15] McMurray JJ (2012). ESC Guidelines for the diagnosis and treatment of acute and chronic heart failure 2012*:* The task force for the diagnosis and treatment of acute and chronic heart failure 2012 of the European Society of Cardiology. Developed in collaboration with the heart failure association (HFA) of the ESC. Eur Heart J.

[16] Feldman D, Pamboukian SV, Teuteberg JJ, Birks E, Lietz K, Moore SA (2013). The 2013 International Society for Heart and Lung Transplantation guidelines for mechanical circulatory support: executive summary. J Heart Lung Transplant..

[17] Yancy CW, Jessup M, Bozkurt B, Butler J, Casey DE, Drazner MH (2013). 2013 ACCF/AHA guideline for the management of heart failure: executive summary: a report of the American College of Cardiology Foundation/American Heart Association task force on practice guidelines. Circulation..

[18] Nishimura RA, Otto CM, Bonow RO, Carabello BA, Erwin JP, Guyton RA (2014). 2014 AHA/ACC guideline for the Management of Patients with Valvular Heart Disease: a report of the American College of Cardiology/American Heart Association task force on practice guidelines. Circulation..

[19] Allen LA, Stevenson LW, Grady KL, Goldstein NE, Matlock DD, Arnold RM (2012). Decision making in advanced heart failure: a scientific statement from the American Heart Association. Circulation..

[20] Fang JC, Ewald GA, Allen LA, Butler J, Westlake Canary CA, Colvin-Adams M (2015). Advanced (stage D) heart failure: a statement from the Heart Failure Society of America guidelines committee. J Card Fail..

[21] Ferrell BR, Temel JS, Temin S, Alesi ER, Balboni TA, Basch EM (2017). Integration of palliative care into standard oncology care: American Society of Clinical Oncology clinical practice guideline update. J Clin Oncol..

[22] Dans M, Smith T, Back A, Baker JN, Bauman JR, Beck AC (2017). NCCN guidelines insights: palliative care, version 2. 2017. J Natl Compr Cancer Netw..

[23] Smith TJ, Temin S, Alesi ER, Abernethy AP, Balboni TA, Basch EM (2012). American Society of Clinical Oncology provisional clinical opinion: the integration of palliative care into standard oncology care. J Clin Oncol..

[24] Rocque GB, Campbell TC, Johnson SK, King J, Zander MR, Quale RM (2015). A quantitative study of triggered palliative care consultation for hospitalized patients with advanced Cancer the results of cohorts 1 and 2 were presented at the 2014 annual assembly of the American Academy of hospice and palliative medicine (AAHPM) and the hospice and palliative nurses association (HPNA). J Pain Symptom Manag..

[25] Adelson K, Paris J, Horton JR, Hernandez-Tellez L, Ricks D, Morrison RS (2017). Standardized criteria for palliative care consultation on a solid tumor oncology service reduces downstream health care use. J Oncol Pract..

[26] Molin Y, Gallay C, Gautier J, Lardy-Cleaud A, Mayet R, Grach MC (2019). PALLIA-10, a screening tool to identify patients needing palliative care referral in comprehensive cancer centers: a prospective multicentric study (PREPA-10). Cancer Medicine..

[27] Brinkman-Stoppelenburg A, et al. The COMPASS study: a descriptive study on the characteristics of palliative care team consultation for cancer patients in hospitals. Eur J Cancer Care. 2019:e13172. 10.1111/ecc.13172 31571338

[28] Temel JS, Greer JA, Muzikansky A, Gallagher ER, Admane S, Jackson VA (2010). Early palliative care for patients with metastatic non-small-cell lung cancer. N Engl J Med..

[29] Hui D, Ross J, Park M, Dev R, Vidal M, Liu D (2020). Predicting survival in patients with advanced cancer in the last weeks of life: how accurate are prognostic models compared to clinicians' estimates?. Palliat Med..

[30] Caraceni A, Lo Dico S, Zecca E, Brunelli C, Bracchi P, Mariani L (2020). Outpatient palliative care and thoracic medical oncology: referral criteria and clinical care pathways. Lung Cancer..

[31] Gemmell R, Yousaf N, Droney J (2020). “Triggers” for early palliative care referral in patients with cancer: a review of urgent unplanned admissions and outcomes. Support Care Cancer..

[32] Hansen MB, Nylandsted LR, Petersen MA, Adsersen M, Rojas-Concha L, Groenvold M (2020). Patient-reported symptoms and problems at admission to specialized palliative care improved survival prediction in 30,969 cancer patients: a nationwide register- based study. Palliat Med..

[33] Sanders SL, Bantum EO, Owen JE, Thornton AA, Stanton AL (2010). Supportive care needs in patients with lung cancer. Psycho-Oncology..

[34] Grudzen CR, Richardson LD, Morrison M, Cho E, Sean MR (2010). Palliative care needs of seriously ill, older adults presenting to the emergency department. Acad Emerg Med..

[35] Glare PA, Semple D, Stabler SM, Saltz LB (2011). Palliative care in the outpatient oncology setting: evaluation of a practical set of referral criteria. J Oncol Pract..

[36] Glare P, Plakovic K, Schloms A, Egan B, Epstein AS, Kelsen D (2013). Study using the NCCN guidelines for palliative care to screen patients for palliative care needs and referral to palliative care specialists. JNCCN..

[37] Hui D, Mori M, Watanabe SM, Caraceni A, Strasser F, Saarto T (2016). Referral criteria for outpatient specialty palliative cancer care: an international consensus. Lancet Oncol..

[38] Bakitas MA, Tosteson TD, Li Z, Lyons KD, Hull JG, Li Z (2015). Early versus delayed initiation of concurrent palliative oncology care: patient outcomes in the ENABLE III randomized controlled trial. J Clin Oncol..

[39] Scandrett KG, Reitschuler-Cross EB, Nelson L, Sanger JA, Feigon M, Boyd E (2010). Feasibility and effectiveness of the NEST13+ as a screening tool for advanced illness care needs. J Palliat Med..

[40] Cohen SR (1997). Validity of the McGill quality of life questionnaire in the palliative care setting: a multi-Centre Canadian study demonstrating the importance of the existential domain. Palliat Med..

[41] Bruera E, Kuehn N, Miller MJ, Selmser P, Macmillan K (1991). The Edmonton symptom assessment system (ESAS): a simple method for the assessment of palliative care patients. J Palliat Care..

[42] Hui D (2016). Referral Criteria for Outpatient Palliative Cancer Care: A Systematic Review. Oncologist.

[43] Harding R, Beynon T, Hodson F, Coady E, Kinirons M, Selman L (2009). Provision of palliative care for chronic heart failure inpatients: how much do we need?. BMC Palliat Care..

[44] Levy WC (2006). The Seattle Heart Failure Model: prediction of survival in heart failure. Circulation.

[45] James T, Offer M, Wilson M, Delyea A, Johnson D (2010). Increasing palliative consults for heart failure inpatients using the Seattle heart failure model. J Hosp Palliat Nurs..

[46] Ng Fat Hing N (2018). Utility of the Seattle Heart Failure Model for palliative care referral in advanced ambulatory heart failure. BMJ Support Palliat Care.

[47] Avula S, LaFata M, Nabhan M, Allana A, Toprani B, Scheidel C (2020). Heart failure mortality prediction using PRISM score and development of a classification and regression tree model to refer patients for palliative care consultation. Int J Cardiol Heart Vasculature..

[48] Campos S, Zhang L, Sinclair E, Tsao M, Barnes EA, Danjoux C (2009). The palliative performance scale: examining its inter-rater reliability in an outpatient palliative radiation oncology clinic. Support Care Cancer..

[49] Green CP, Porter CB, Bresnahan DR, Spertus JA (2000). Development and evaluation of the Kansas City cardiomyopathy questionnaire: a new health status measure for heart failure. J Am Coll Cardiol.

[50] Abernethy AP, Shelby-James T, Fazekas BS, Woods D, Currow DC. The Australia-modified Karnofsky Performance Status (AKPS) scale: a revised scale for contemporary palliative care clinical practice. BMC Palliat Care. 2005;4(7). 10.1186/1472-684X-4-7 PMC1308820 16283937

[51] Ezekowitz JA, Thai V, Hodnefield TS, Sanderson L, Cujec B (2011). The correlation of standard heart failure assessment and palliative care questionnaires in a multidisciplinary heart failure clinic. J Pain Symptom Manag..

[52] Greener DT, Quill T, Amir O, Szydlowski J, Gramling RE (2014). Palliative care referral among patients hospitalized with advanced heart failure. J Palliat Med..

[53] Campbell RT (2018). Which patients with heart failure should receive specialist palliative care?. Eur J Heart Fail..

[54] Kane PM, Murtagh FEM, Ryan KR, Brice M, Mahon NG, McAdam B (2018). Strategies to address the shortcomings of commonly used advanced chronic heart failure descriptors to improve recruitment in palliative care research: a parallel mixed- methods feasibility study. Palliat Med..

[55] Roch C, et al. Utility of the integrated palliative care outcome scale (IPOS): a cross-sectional study in hospitalised patients with heart failure. Eur J Cardiovasc Nurs. 2020:1474515120919386. 10.1177/1474515120919386 32370552

[56] Unroe KT, Greiner MA, Hernandez AF, Whellan DJ, Kaul P, Schulman KA (2011). Resource use in the last 6 months of life among medicare beneficiaries with heart failure, 2000-2007. Arch Intern Med..

[57] Kheirbek RE, Fletcher RD, Bakitas MA, Fonarow GC, Parvataneni S, Bearden D (2015). Discharge hospice referral and lower 30-day all-cause readmission in Medicare beneficiaries hospitalized for heart failure. Circ Heart Fail..

[58] Webster K, Cella D, Yost K. The Functional Assessment of Chronic Illness Therapy (FACIT) Measurement System: properties, applications, and interpretation. Health Qual Life Outcomes. 2003;1(79). 10.1186/1477-7525-1-79 PMC317391 14678568

[59] Rogers JG, Patel CB, Mentz RJ, Granger BB, Steinhauser KE, Fiuzat M (2017). Palliative Care in Heart Failure: the PAL-HF randomized, controlled clinical trial. J Am Coll Cardiol..

